# When self-control is no longer a protective factor: the moderating effect of institutionalized identity on attitudes toward artificial intelligence

**DOI:** 10.3389/fpsyg.2026.1851183

**Published:** 2026-07-23

**Authors:** Ying Long, Yuan Zhou, Die Luo, Ming Chang

**Affiliations:** 1 School of Psychology, Shaanxi Normal University, Xi'an, China; 2 Shaanxi Provincial Key Laboratory of Behavior and Cognitive Neuroscience, Xi'an, China; 3 Aviation Human Factors and Artificial Intelligence Psychology Research Group, School of Psychology, Shaanxi Normal University, Xi'an, China

**Keywords:** attitudes toward artificial intelligence, functional contextualization, high-responsibility occupations, institutionalized identity, self-control

## Abstract

As artificial intelligence (AI) is increasingly applied in high-risk industries, understanding the attitudes of different occupational groups toward AI and their underlying psychological correlates has become an important research issue. Existing studies typically conceptualize self-control as a psychological trait with universal adaptive value, suggesting that it helps buffer “individuals” negative attitudes toward new technologies; however, this assumption is primarily based on samples from the general population. Drawing on a situated perspective of psychological functioning, the present cross-sectional study examined whether institutionalized identity moderates the relationship between self-control and negative attitudes toward AI, using a convenience sample of 289 participants (179 university students, 76 pilots, and 34 flight instructors). Negative AI attitudes were assessed with the Negative Attitudes toward Artificial Intelligence Scale (NARS), which captures concerns and reservations toward AI rather than acceptance, trust, reliance, or behavioral adoption. Results showed that among university students, self-control was stably and negatively associated with negative AI attitudes; among pilots, this relationship was no longer significant; and among flight instructors, a non-significant positive trend was observed, which—given the small subgroup size (*n* = 34)—should be regarded as a preliminary, exploratory finding rather than a confirmed effect. These results are consistent with the interpretation that the functional priority of self-control may shift from emotional buffering toward risk vigilance in high-responsibility occupational contexts; however, because the proposed underlying psychological processes (e.g., capability anxiety vs. responsibility-based caution) were not directly measured, this account is offered as a plausible interpretation rather than a mechanism demonstrated by the present data. In addition, the study introduces a dual-dimensional “value–dynamics” analytical framework as a conceptual extension intended to guide future research, since the dynamics-orientation dimension was not directly assessed in the present design. Overall, the findings provide preliminary evidence for the context-dependent nature of psychological trait effects and offer tentative implications for AI governance and occupational training in high-responsibility professional settings: AI integration programs and attitude-related interventions may need to be tailored to the distinct responsibility structures and psychological orientations of different occupational groups, rather than assuming universal effects derived from general-population research.

## 1 Introduction

AI technology is increasingly embedded in multiple critical domains of social functioning, reshaping human work patterns, decision-making processes, and the allocation of responsibility. The aviation sector is particularly illustrative: from autopilot systems and intelligent flight-path planning to predictive maintenance and smart air traffic management, AI is progressively entering the core of flight decision-making and safety assurance (Ali et al., 2025; Kistan et al., 2017). International civil aviation organizations and national regulators have identified AI as a key direction for the digital transformation of aviation (European Aviation Safety Agency, 2020). However, advances in technical capability do not automatically translate into full acceptance at the operational level. Individuals and groups differ substantially in their attitudes toward AI (Stein et al., 2024), and these differences affect not only technology adoption behaviors but also the safety and effectiveness of human–machine collaboration (Gillath et al., 2021). In high-risk industries, practitioners' attitudes toward AI are often embedded in deeper considerations of responsibility attribution, risk consequences, and the boundaries of professional judgment. Ignoring these psychological and socio-structural factors may impede the effective deployment of AI, and in certain contexts could even introduce new systemic risks (Qi et al., 2024). Understanding the differences in AI attitudes across occupational groups and their psychological bases (Küper and Krämer, 2025) has therefore become an important agenda item in AI social governance.

Existing research on the psychological mechanisms underlying AI attitudes often contains an insufficiently examined assumption: that individual psychological traits function consistently across different occupational and responsibility contexts. Is this premise valid? Do psychological regularities discovered in general populations apply equally in high-responsibility occupational settings?

AI attitudes refer to individuals‘ overall evaluative orientations toward AI technology and its applications, typically comprising three interrelated components: cognitive, affective, and behavioral-intentional (Schepman and Rodway, 2020). The cognitive component involves beliefs about AI's capabilities, reliability, and potential consequences; the affective component reflects emotional experiences when confronted with AI, such as feelings of security, anxiety, or perceived threat; the behavioral-intentional component encompasses tendencies to use, rely on, or avoid AI systems. Early research largely situated AI attitudes within the Technology Acceptance Model (TAM) or the Unified Theory of Acceptance and Use of Technology (UTAUT) framework (Davis, 1989; Venkatesh et al., 2003), focusing on how technology-attribute variables such as perceived usefulness and perceived ease of use influence attitudes and usage intentions. As AI has evolved from a tool-support technology into a system with autonomous, decision-participating capabilities, the limitations of these traditional acceptance models have become apparent (Ibrahim et al., 2025). Compared with general information technology, AI is more likely to trigger psychological concerns around autonomy, control, and responsibility attribution (Schaefer et al., 2016; Xu et al., 2024), and recent scholarship has converged on trust as the central psychological pivot of human–AI engagement, with appropriately calibrated trust depending on individuals' technology self-efficacy, perceived risk, and dispositions toward automation (Küper and Krämer, 2025).

In recent years, research has increasingly shifted from a single technological-attribute perspective toward multidimensional exploration of psychological mechanisms, with particular attention to individual-difference factors (Huang and Li, 2024). Studies have found that personality traits, cognitive styles, and risk preferences are all associated with AI attitudes (Sindermann et al., 2021; Stein et al., 2024). The most recent evidence further refines this picture: Big Five traits, gender, and age predict positive attitudes toward AI in ways that are distinguishable from attitudes toward general technology (Grassini et al., 2025), and trait-based determinants have been shown to shape AI evaluations even in highly trained professional samples such as medical students (Qi et al., 2026). Building on this individual-difference turn, broader social-psychological factors also matter: Li et al. (2026), advance online publication demonstrated that subjective social class influences AI attitudes through social mobility beliefs, indicating that AI attitudes are embedded in broader social-psychological structures. Across this body of work, however, participants are still predominantly drawn from general or student populations. For these groups, the primary psychological task when facing AI is typically managing the anxiety induced by technological uncertainty (Meuter et al., 2003), and psychological resources such as self-control (Baumeister et al., 2007) naturally function to buffer anxiety and facilitate rational evaluation. These studies thus implicitly assume cross-situational consistency in the attitudinal effects of psychological traits.

The situation may differ for high-responsibility professionals such as pilots and physicians (Dean et al., 2024). Practitioners in high-risk domains develop stable professional identities and risk-perception frameworks through prolonged occupational socialization (Casner and Schooler, 2014), and their attitudes toward automated systems are often embedded in deep concerns about responsibility attribution, the boundaries of professional judgment, and system safety (Parasuraman and Riley, 1997; Schaefer et al., 2016). Aviation-specific evidence reinforces this point: research on student pilots indicates that AI-related anxieties, perceived trustworthiness, and adaptation processes interact in patterns that diverge from those typically observed in general-population samples (Çeken et al., 2025). If the core psychological tasks that different occupational groups face when confronting AI differ fundamentally, it is worth investigating whether the same psychological trait can still serve the same function across these tasks.

Self-control is broadly defined as the ability to regulate impulses, delay immediate gratification, and maintain goal-directed behavior, and is a widely studied individual-difference variable in psychology (Tangney et al., 2018). Extensive research has shown that individuals with higher self-control tend to perform better academically, adapt more effectively in occupational settings, engage in healthier behaviors, and show greater adherence to social norms (De Ridder et al., 2012; Duckworth and Seligman, 2005). In technology contexts, self-control has typically been conceptualized as a psychological resource that helps reduce uncertainty, buffer anxiety, and promote rational decision-making (Baumeister et al., 2007). Existing research commonly predicts a negative relationship between self-control and negative attitudes toward AI: individuals with higher self-control are more likely to suppress instinctive rejection of new technology, rationally weigh benefits against risks, and consequently form fewer negative attitudes. This expectation is consistent with the uncertainty-reduction pathway emphasized in TAM (Davis, 1989; Venkatesh et al., 2003). These findings rest on the important theoretical premise that the function of self-control is consistent across contexts. Recent scholarship has begun to question this premise. Situated personality research holds that the behavioral expression and functional meaning of psychological traits often depend on specific contextual conditions (Fleeson and Jayawickreme, 2015). Uziel and Baumeister (2017) found that, in certain highly demanding situations, the effects of self-control may change and even exhibit patterns opposite to those observed in ordinary contexts. By extension, in high-responsibility occupational settings, the dominant function of self-control may shift from buffering anxiety and facilitating acceptance to maintaining vigilance and exercising cautious evaluation—not a negation of its adaptive value, but a situational reorientation of its functional direction. This reasoning suggests that the relationship between self-control and negative AI attitudes may manifest differently across occupational groups.

To account for the contextual variation in self-control's functioning, the present study introduces the concept of “institutionalized identity.” Role theory holds that social roles carry specific behavioral expectations and normative constraints, and that individuals gradually internalize these norms as they take on roles (Biddle, 1986). Institutional theory further emphasizes that highly institutionalized domains systematically shape individual cognitive frameworks and behavioral patterns (DiMaggio and Powell, 1983). Integrating these perspectives, the present study defines institutionalized identity as the stable identity that individuals form through prolonged occupational socialization by internalizing specific institutional norms. This concept is distinct from simple occupational categorization: whereas occupational identity is a surface-level group label, institutionalized identity emphasizes the deep internalization of professional norms and responsibility structures, and the resulting functional reconfiguration of psychological processes.

This perspective is complementary to, but distinct from, several existing frameworks in human–AI research. Technology-acceptance models (TAM/UTAUT) explain AI attitudes primarily as a function of perceived technology properties (Davis, 1989; Venkatesh et al., 2003); human–AI trust research focuses on calibrated trust as a psychological state shaped by system characteristics, dispositional trust propensity, and prior experience (Küper and Krämer, 2025; Schaefer et al., 2016); and technology-anxiety and approach–avoidance accounts emphasize individual emotional reactions to technology uncertainty (Meuter et al., 2003). What these frameworks share is a tendency to treat psychological factors—trust, anxiety, risk perception, control beliefs—as relatively portable across contexts, with occupation entering at most as a background covariate or sampling stratum. The institutionalized identity lens differs in that it treats the occupational role itself as a structural moderator of how psychological resources are mobilized: the same trait may be recruited to serve emotional regulation in one identity and risk monitoring in another, because the responsibility structures embedded in different institutional roles reorganize what counts as adaptive psychological functioning. This lens is particularly appropriate for AI attitudes in high-responsibility settings, where occupational accountability is not merely a background variable but a defining feature of the psychological situation.

Different institutionalized identities thus correspond to different responsibility structures and psychological tasks, and the present design samples three groups arrayed along a continuum of institutional embeddedness to test whether the functional meaning of self-control shifts as a function of this continuum. University students occupy the low-end anchor of the continuum: they are in the stage of emerging adulthood with identity still under exploration and no formal professional responsibilities yet assumed (Arnett, 2000), and their primary psychological task when confronting new technology is typically managing uncertainty-induced anxiety. The student group is included not because they are practitioners of high-risk work, but because they provide the theoretically meaningful baseline against which the institutionalized-identity hypothesis can be tested: only by comparing the self-control–attitude link in a no-responsibility group against the same link in high-responsibility groups can one assess whether the link is genuinely identity-dependent rather than uniform across populations. Pilots occupy the middle position: after systematic professional training, they have formed stable professional identities and a strong safety culture, with the core responsibility of ensuring flight safety (Biedermann et al., 2024); when facing AI they must evaluate its implications for professional judgment and operational autonomy. Flight instructors occupy the high-end anchor, bearing not only flight-safety responsibilities but also the duty of cultivating trainees and transmitting professional norms (Fanjoy and Gao, 2011); their considerations of AI may involve broader concerns about professional ethics and intergenerational continuity.

From this perspective, self-control may serve different functions in different institutionalized identities. For university students, self-control primarily operates to inhibit impulsive reactions and regulate negative emotions, thereby facilitating rational appraisal of new phenomena (Baumeister et al., 2007)—consistent with classic theoretical expectations. For pilots and flight instructors, however, self-control may be more readily mobilized to maintain sensitivity to risk and vigilance about the boundaries of responsibility (Warm et al., 2008). In this case, individuals with higher self-control may not exhibit lower negative AI attitudes; rather, they may display a more cautious evaluative orientation. The aviation domain provides an ideal research setting for testing these propositions (Dong and Fang, 2021). Pilots and flight instructors develop highly stable professional norms and risk-perception frameworks through prolonged occupational socialization (Casner and Schooler, 2014), with salient institutionalized identity characteristics. At the same time, AI applications in aviation are expanding rapidly, and practitioners' attitudes toward AI carry direct practical significance. Using aviation practitioners as a sample thus facilitates examination of how institutionalized identity may moderate the relationship between self-control and AI attitudes.

In summary, the present study aims to investigate whether the relationship between self-control and negative AI attitudes varies by institutionalized identity, and to preliminarily explore the psychological mechanisms underlying such variation. Through this investigation, the study seeks to provide a new perspective for understanding the psychological bases of AI attitudes and to offer reference points for AI governance strategies across different occupational groups. The study also examines whether this variation might involve changes in the direction of effects, not merely in effect magnitude. Based on the foregoing analysis, the following hypotheses are proposed:

Hypothesis 1 (H1): In the overall sample, self-control will be significantly and negatively associated with negative AI attitudes.
Hypothesis 2 (H2): Institutionalized identity will moderate the relationship between self-control and negative AI attitudes.
Hypothesis 3 (H3): Relative to university students, the negative relationship between self-control and negative AI attitudes will be attenuated in high-responsibility occupational groups (pilots, flight instructors).

## 2 Materials and methods

### 2.1 Participants

A total of 289 participants were recruited, comprising three groups: 179 university students, 76 pilots, and 34 flight instructors. The university student sample was drawn from regular higher education institutions; the pilot and flight instructor samples were recruited from the same airline. Mean ages were *M* = 21.56 years(*SD* = 1.81), *M* = 29.33 years(*SD* = 3.74), and *M* = 43.03 years(*SD* = 6.69)for the three groups, respectively. The overall sample included 171 male participants (59.2%). The proportion of female participants was higher in the student group (65.4%), while pilots and flight instructors were predominantly male—a distribution consistent with the actual gender composition of the aviation industry. In terms of flight experience, most pilots had 4–7 years and most flight instructors had 15 or more years. All participants volunteered to take part in the study and provided written informed consent prior to beginning the survey. In all analyses, occupational identity was operationalized as a linear variable reflecting the cumulative depth of professional socialization and occupational responsibility, coded as 0 (university students: no formal occupational responsibility), 1 (pilots: 1–10 years of high-responsibility occupational experience), and 2 (flight instructors: 10 or more years of occupational experience plus instructional responsibility). This coding scheme captures the progressive internalization of institutional norms across groups. This linear operationalization is grounded in the theoretical construct of institutionalized identity as defined in the present study. The three groups represent successive stages of occupational socialization, each involving a qualitative increase in both formal responsibility and norm internalization: university students operate outside formal occupational institutions and bear no professional accountability; pilots undergo systematic vocational training and assume direct safety-critical responsibilities; flight instructors additionally bear supervisory and norm-transmission duties. The ordering thus reflects the cumulative depth of institutional embeddedness rather than an arbitrary rank. Nevertheless, the equal-interval assumption inherent in linear coding may not fully hold. Accordingly, all substantive conclusions are interpreted in conjunction with the pattern of simple slopes for each group individually, and future work employing dummy-coded or polynomial contrasts could further examine the precise functional shape of the identity-by-self-control interaction.

Methodologically, the present design is a cross-sectional, between-groups moderation study. The three occupational groups serve as theoretically meaningful representatives of distinct positions along the institutionalized-identity continuum, but they are not matched on age, gender composition, professional experience, or aviation/AI exposure; covariation among these variables and institutionalized identity is a structural feature of the present sample. Accordingly, the analyses are interpreted as a moderation test using three theoretically informative but imperfectly comparable groups, with age and gender entered as covariates in robustness checks (see the Robustness Check section). Implications of this design for causal inference and generalizability are addressed in the Limitations section.

### 2.2 Measures

#### 2.2.1 Self-control scale

Self-control was assessed using the Chinese version of the Self-Control Scale revised by Tan and Guo (2008). This scale derives from the original Self-Control Scale (SCS) developed by Tangney et al. (2018) and was adapted and validated for the Chinese cultural context. The original scale contains 36 items; a 13-item brief version (Brief Self-Control Scale, BSCS) was subsequently developed. The Chinese version contains 19 items and yields a five-factor structure: impulse control (e.g., “I get carried away by my feelings”), healthy habits (e.g., “It is hard for me to break bad habits”), resisting temptation (e.g., “Sometimes I can't stop myself from doing something, even if I know it is wrong”), focused work (e.g., “Sometimes I get distracted by fun things and fail to complete my tasks on time”), and recreational moderation (e.g., “I do things that bring me pleasure even if they are bad for me”). Items were rated on a 5-point Likert scale (1 = strongly disagree, 5 = strongly agree), with some items reverse-scored; higher scores indicate higher self-control. Prior research has demonstrated satisfactory psychometric properties of this scale in Chinese samples (Unger et al., 2016). In the present study, the overall Cronbach's α was 0.93; subscale α values were: impulse control = 0.91, healthy habits = 0.80, resisting temptation = 0.78, focused work = 0.65, and recreational moderation = 0.70.

#### 2.2.2 Negative attitudes toward artificial intelligence scale

Negative attitudes toward AI were assessed using the Negative Attitudes toward Artificial Intelligence Scale (NARS; Nomura et al., 2006). Although the NARS was originally developed to measure negative attitudes toward robots, it has since been widely adapted and validated in AI-related research contexts. Its item content explicitly references both robots and AI (e.g., “I feel very nervous standing in front of a robot or AI”), and prior studies have confirmed acceptable construct validity when applied to AI attitudes in non-robotic settings (e.g., Sindermann et al., 2021; Stein et al., 2024). This 11-item scale measures individuals' negative evaluative orientations toward AI technology and its social consequences, covering concerns about overreliance on AI (e.g., “I think it is bad to rely too much on artificial intelligence”), distrust of AI judgment and decision-making, worry about potential social consequences of AI, and tension when interacting with AI (e.g., “I feel very nervous standing in front of a robot or AI”). Items were rated on a 7-point Likert scale (1 = strongly disagree, 7 = strongly agree), with some items reverse-scored; higher scores indicate more negative attitudes. Cronbach's α for this scale in the present study was 0.85.

It should be noted that the NARS, as used here, indexes the negative-evaluation dimension of AI attitudes—concerns, distrust, and discomfort—rather than positive valuation, calibrated trust, behavioral reliance, or actual AI use. The present study therefore assesses one specific dimension of the broader AI-attitude space, and conclusions drawn from the NARS apply to that dimension only.

#### 2.2.3 Demographic variables

Demographic variables included gender, age, occupational identity, highest educational level, years of flight or teaching experience, and AI exposure. AI exposure was assessed with a single item asking whether the respondent's organization (university or employer) had introduced AI-related systems or tools (yes/no/unsure); this item indexes organizational AI exposure rather than individual frequency of AI use. Occupational identity (university student, pilot, flight instructor) served as the key grouping variable.

### 2.3 Procedure

Data were collected via an online survey platform. Participants accessed the survey by scanning a QR code or clicking a study link; after reading and consenting to the informed consent form, they completed the questionnaire in the following order: demographic information, the Self-Control Scale, and the NARS. Total completion time was approximately 8 min. The study was conducted in accordance with the Declaration of Helsinki. Because the research involved only an anonymous, non-interventional online questionnaire administered to competent adult volunteers, did not collect identifying information or sensitive personal data, and posed no foreseeable risk to participants, it qualified as exempt from formal Institutional Review Board review under the applicable national and institutional research-ethics regulations. All participants were informed of the purpose, voluntary nature, and anonymity of the study; provided written informed consent prior to participation; were free to withdraw at any point without consequence; and had all data anonymized and stored confidentially.

Data quality procedures differed by group. For the pilot and flight instructor samples, all questionnaires were collected in full with no cases excluded at the individual level. A small number of participants showed obvious uniform response patterns (e.g., selecting the same option repeatedly across an entire scale); given the scarcity of this professional sample and its occupational homogeneity, the affected items were replaced with the within-group mean value to minimize the potential influence of anomalous responses on statistical results, without affecting the overall distribution of the scale. This approach was preferred over listwise deletion because excluding even a small number of cases from the professional groups would have substantially reduced statistical power and further compromised the analysis of an already underpowered subgroup (*n* = 34 for flight instructors). Within-group mean imputation is a recognized conservative strategy in small-sample occupational research when complete-case removal is not viable (cf. Schafer and Graham, 2002). For the student sample, 185 questionnaires were initially collected; after applying pre-established data quality criteria (exclusion of monotone response patterns, excessively short completion times, and otherwise anomalous patterns), 179 participants were retained for analysis.

## 3 Results

### 3.1 Sensitivity power analysis

Because the three groups differed substantially in size, we conducted sensitivity power analyses (*F*-test framework for multiple regression; Cohen, 1988; α = 0.05) to characterize the smallest effects each test could reliably detect. For the focal interaction in the full sample (*N* = 289, with both lower-order terms partialled), the observed effect was small (*f*^2^ = 0.02; Δ*R*^2^ = 0.018), and the design afforded approximately 70% power to detect an effect of this magnitude; the minimum effect detectable at 80% power was *f*^2^ = 0.028 (Δ*R*^2^ ≈ 0.023). Thus, although the interaction was statistically significant (*p* = 0.014), the moderation test was powered to detect only small-or-larger interaction effects. Within-group sensitivity was more limited: at 80% power (two-tailed, α = 0.05), the flight-instructor subgroup (*n* = 34) could reliably detect only standardized slopes of |*r*| ≥ 0.44 (medium-to-large effects), and the pilot subgroup (*n* = 76) only |*r*| ≥ 0.31. Because the observed instructor slope was small-to-moderate (|*r*| ≈ 0.23), this subgroup was substantially underpowered, reinforcing our treatment of the positive instructor slope as a preliminary, exploratory pattern rather than a confirmed effect.

### 3.2 Common method bias

Common method bias was evaluated using three complementary approaches. First, Harman's single-factor test (an unrotated exploratory factor analysis of all measured items) revealed that the first extracted factor accounted for 31.5% of the total variance, below the 40% threshold (Podsakoff et al., 2003). Second, to compare measurement-model structures on a common set of indicators, we constructed item parcels for each construct and, using these parcels, contrasted a single-factor model with a two-factor model distinguishing self-control from negative AI attitudes. The single-factor model, in which all parcels loaded on one latent factor, fit the data poorly (χ^2^*/df* = 7.56, *CFI* = 0.49, *TLI* = 0.45, *RMSEA* = 0.15), whereas the two-factor model fit well (*CFI* = 0.996, *RMSEA* = 0.05); the two-factor model fit significantly better than the single-factor model (Δχ^2^(1) = 708.6, *p* < 0.001). Third, we added an unmeasured latent method factor orthogonal to the substantive factors; this common method factor accounted for an average of 17% of indicator variance, below the conventional threshold of concern. Together, these results indicate that common method bias is unlikely to account for the observed relationships. Moreover, the study's central finding is a cross-group interaction, which shared method variance tends to attenuate rather than inflate (Siemsen et al., 2010), and the group-specific divergence in slopes could not plausibly arise as a uniform method artifact.

### 3.3 Descriptive statistics and correlation analysis

Descriptive statistics and bivariate correlations among the main variables are presented in Table 1. Occupational identity was significantly positively correlated with negative AI attitudes (*r* = 0.32, *p* < 0.001), indicating that pilots and flight instructors held more negative attitudes toward AI than did university students. Self-control was significantly negatively correlated with negative AI attitudes (*r* = −0.32, *p* < 0.001), such that higher self-control was associated with fewer negative attitudes. Additionally, occupational identity was significantly negatively correlated with self-control (*r* = – 0.34, *p* < 0.001), reflecting systematic group differences in self-control levels.

**Table 1:** Descriptive statistics and bivariate correlations among main variables (*N* = 289).

Variable	*M*	*SD*	1	2	3
Occupational identity	0.50	0.70	—		
Self-control	3.05	0.78	−0.34^***^	—	
Negative AI attitudes	3.58	0.97	0.32^***^	−0.32^***^	—

^***^p < 0.001.

Group-level descriptive statistics are presented in Table 2. University students showed the highest self-control (*M* = 3.28, *SD* = 0.85); pilots (*M* = 2.65, *SD* = 0.45) and flight instructors (*M* = 2.70, *SD* = 0.49) showed lower levels. Regarding negative AI attitudes, university students scored lowest (*M* = 3.35, *SD* = 1.03), pilots intermediate (*M* = 3.86, *SD* = 0.75), and flight instructors highest (*M* = 4.16, *SD* = 0.58). One-way ANOVA revealed significant group differences for both self-control, *F*_(2, 286)_ = 24.72, *p* < 0.001, and negative AI attitudes, *F*_(2, 286)_ = 16.09, *p* < 0.001.

**Table 2:** Descriptive statistics by occupational group.

Variable	Students (*n* = 179)	Pilots (*n* = 76)	Instructors (*n* = 34)	*F*
Self-control	3.28 ± 0.85	2.65 ± 0.45	2.70 ± 0.49	24.72^***^
Negative AI attitudes	3.35 ± 1.03	3.86 ± 0.75	4.16 ± 0.58	16.09^***^

^***^p < 0.001.

**Figure 1:**
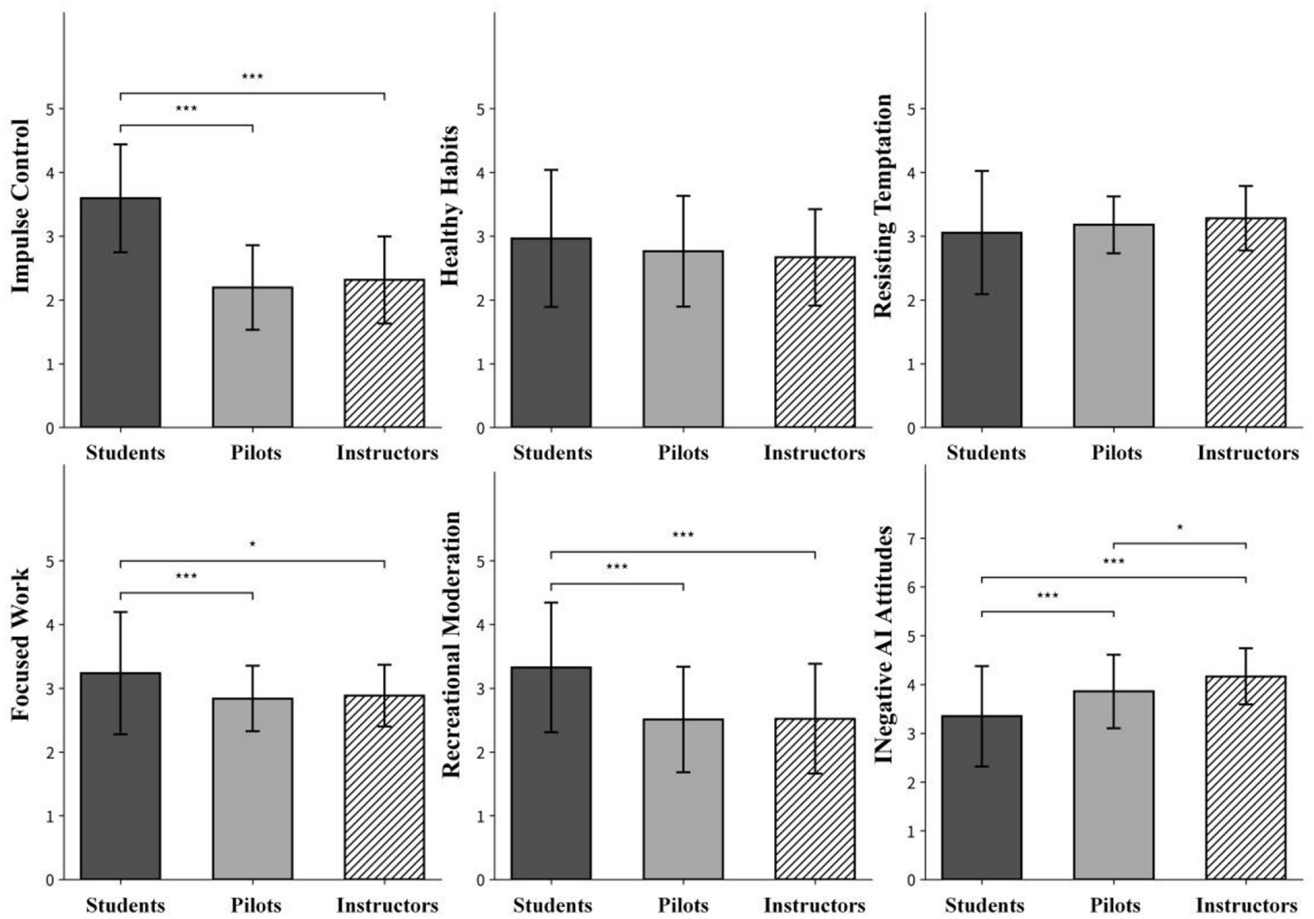
Subscale score comparisons across identity groups. Error bars represent ±1 *SD*. Asterisks denote significance from uncorrected pairwise *t*-tests: *p < 0.05, ***p < 0.001.

To provide a more comprehensive understanding of group differences in self-control, supplementary analyses compared the five self-control subscale scores across groups (see Figure 1). Significant group differences emerged for impulse control, focused work, and recreational moderation, whereas healthy habits and resisting temptation showed no significant differences. This pattern suggests that different institutionalized identities may produce differentiated effects at distinct functional levels of self-control, providing a foundation for interpreting the subsequent moderation effects.

### 3.4 Moderation analyses

The bivariate correlation confirmed that self-control was significantly negatively correlated with negative AI attitudes (*r* = –.32, *p* < 0.001), supporting Hypothesis 1. The following analyses further tested the moderating role of institutionalized identity.

#### 3.4.1 Moderation test

Hierarchical regression was used to test whether occupational identity moderates the relationship between self-control and negative AI attitudes. Self-control was mean-centered prior to analysis. Step 1 entered the main effects of self-control and occupational identity; Step 2 added the interaction term. Results showed that the interaction term significantly predicted negative AI attitudes, *B* = 0.35, *SE* = 0.14, *t* = 2.47, *p* = 0.014, 95% CI [0.07, 0.62], Δ*R*^2^ = 0.02, supporting Hypothesis 2 (see Table 3).

**Table 3:** Hierarchical regression models.

Variable	*B*	*SE*	*t*	*P*	*95% CI*	*R^2^*
Model 1						0.152
Constant	3.42	0.07	51.76	< 0.001	[3.29, 3.55]	
Self-control	−0.30	0.07	−4.21	< 0.001	[−0.44, −0.16]	
Identity	0.32	0.08	3.99	< 0.001	[0.16, 0.48]	
Model 2						0.170
Constant	3.43	0.07	52.22	< 0.001	[3.30, 3.56]	
Self-control	−0.37	0.08	−4.86	< 0.001	[−0.52, −0.22]	
Identity	0.42	0.09	4.71	< 0.001	[0.24, 0.59]	
Self-control × identity	0.35	0.14	2.47	0.014	[0.07, 0.62]	

Δ*R*^2^ = 0.018, Δ*F*_(1, 285)_ = 6.11, *p* = 0.014.

#### 3.4.2 Simple slope analysis

**Figure 2:**
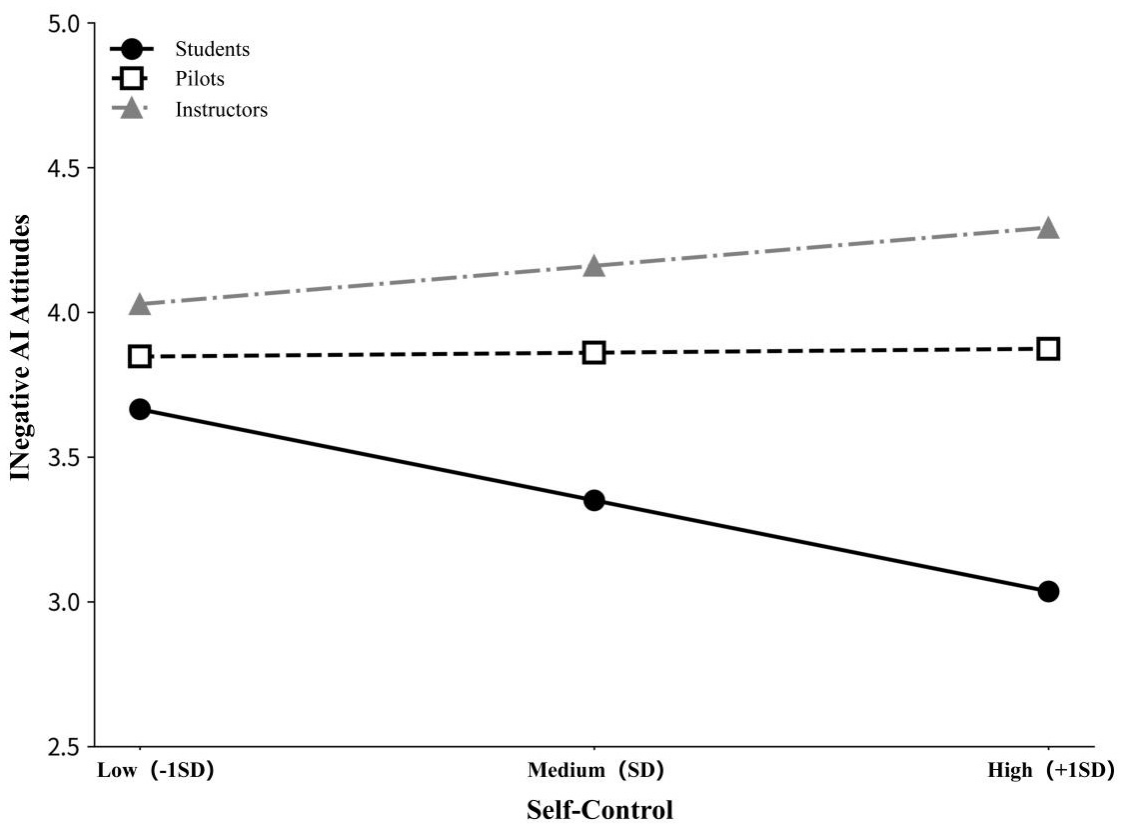
Moderating effect of occupational identity on the relationship between self-control and negative AI attitudes. Lines represent within-group regression lines evaluated at each group's mean ±1 *SD* of self-control.

To further interpret the interaction, simple slopes for the prediction of negative AI attitudes by self-control were examined within each occupational group (see Table 4 and Figure 2).

**Table 4:** Simple slopes for the prediction of negative ai attitudes by self-control across groups.

Group	*n*	*B*	*SE*	*t*	*p*	*95% CI*
Students	179	−0.37	0.09	−4.32	< 0.001	[−0.54, −0.20]
Pilots	76	0.03	0.20	0.16	0.874	[−0.36, 0.42]
Instructors	34	0.27	0.20	1.32	0.197	[−0.15, 0.68]

Test of between-group slope differences: *F*_(2, 283)_ = 3.08, *p* = 0.048.

Self-control significantly and negatively predicted negative AI attitudes among university students (*B* = −0.37, *p* < 0.001). This effect was no longer significant among pilots (*B* = 0.03, *p* =.874). Among flight instructors, a positive trend emerged that did not reach statistical significance (*B* = 0.27, *p* = 0.197). The test of between-group slope differences was significant, *F*_(2, 283)_ = 3.08, *p* = 0.048, indicating that the predictive role of self-control is meaningfully identity-dependent, supporting Hypothesis 3.

#### 3.4.3 Robustness check

The interaction was robust to the inclusion of additional covariates. It remained significant after controlling for age and gender (*B* = 0.34, *p* = 0.018), for educational level (*B* = 0.34, *p* = 0.015), and for organizational AI exposure (*B* = 0.33, *p* = 0.019), and it also remained significant in a model that included all four covariates simultaneously (*B* = 0.31, *p* = 0.028); across all models the direction of the group-specific simple slopes was unchanged. Educational level was coded ordinally (1 = senior high school, 2 = junior college, 3 = bachelor‘s, 4 = master's, 5 = doctorate). Contrary to the expectation that education would be redundant with occupational identity, the two were nearly uncorrelated (*r* = –.04), and collinearity diagnostics were excellent for all predictors (all VIF ≤ 1.14; education VIF = 1.00); educational level did not itself predict negative AI attitudes (*B* = 0.11, *p* =.36). To ensure that the interaction did not depend on the assumptions of ordinary least squares regression, we re-examined it using four additional analytic approaches, and it was robust across all of them: heteroskedasticity-consistent (HC3) standard errors (*B* = 0.35, robust *SE* = 0.11, *p* =.001); a nonparametric case-resampling bootstrap with 10,000 replications (*B* = 0.34, 95% *CI* [0.13, 0.55], *p* =.002); a permutation test with 10,000 permutations of self-control (*p* < .0001); and robust (Huber M-estimator) regression (*B* = 0.39, *p* =.006). A multi-group test confirmed that the three group-specific slopes differed significantly (Wald *F*(2, 283) = 3.08, *p* =.048). Notably, the robust and resampling-based methods yielded smaller standard errors than OLS, indicating that the original OLS test was, if anything, conservative. A supplementary analysis examining the five self-control subscales across occupational groups revealed that all five subscales significantly predicted negative AI attitudes in the student group, whereas none did so in the pilot or instructor groups. These findings are discussed further below.

## 4 Discussion

The present study found that the relationship between self-control and negative AI attitudes appears to vary by institutionalized identity. Among university students, self-control and negative AI attitudes showed a stable negative association consistent with prior research; among pilots, this relationship was substantially attenuated and no longer statistically significant; and among flight instructors, a positive trend emerged that did not reach statistical significance. Given the limited subgroup size (*n* = 34), the instructor finding should be regarded as preliminary and exploratory rather than as confirmed evidence of a directional reversal—a point we return to below and in the Limitations section. The significant test of between-group slope differences nonetheless indicates that the predictive role of self-control is meaningfully identity-dependent. The theoretical significance of this finding lies not in re-confirming a link between self-control and technology attitudes, but in pointing to its possible situational boundaries: the implicit assumption in prior research of cross-situational consistency in self-control's functioning may not extend cleanly to high-responsibility occupational contexts. Previous studies on AI attitudes, largely based on general-population samples, have found that psychological resources such as self-control are associated with more positive technology attitudes (Sindermann et al., 2021; Tangney et al., 2018). The present results suggest that this association may not be robust in high-responsibility occupational settings: the attitudinal effects of psychological traits may have situational boundaries, and their specific manifestations may depend on the responsibility structure and occupational role in which the individual is embedded.

### 4.1 Situational shift in self-control function: from “Emotional Buffering” to “Risk Vigilance”

To interpret these statistical results, we offer an explanation in terms of a situational shift in self-control's function. In the student group, self-control may primarily serve an “emotional buffering” function. It should be emphasized at the outset that the proposed underlying psychological processes—capability-anxiety regulation in the student group, and responsibility-based vigilance in the high-responsibility groups—were not directly measured in this study. The interpretation that follows is therefore offered as a plausible account of the observed group differences in slopes rather than as a mechanism demonstrated by the present data; direct empirical testing of these processes (e.g., via state-anxiety, perceived professional responsibility, or vigilance measures) is an important task for future work. With this caveat in mind, we proceed to outline the situated account that, in our view, best organizes the present pattern of results. University students are in a period of identity exploration (Arnett, 2000), and their primary psychological challenge when confronting AI—a novel technology—is managing uncertainty-induced anxiety (Meuter et al., 2003). In this context, individuals with higher self-control are better able to suppress impulsive reactions, regulate negative emotions, and maintain rational evaluation, thereby forming fewer negative attitudes (Tangney et al., 2018)—a pattern consistent with classic self-control theory (Baumeister et al., 2007).

The situation differs for high-responsibility professionals such as pilots and flight instructors, who have completed occupational socialization and established stable institutionalized identities. When these individuals confront AI, their core concern may have shifted from “Can I adapt to this technology?” to “How will this technology affect my professional judgment, the boundaries of my responsibility, and safety assurance?” (Schaefer et al., 2016). In this context, the functional priority of self-control may change (Uziel and Baumeister, 2017): risk vigilance, rather than emotional buffering, becomes the dominant function (Warm et al., 2008). Individuals with higher self-control may therefore tend not toward lower negative AI attitudes but rather toward a more cautious evaluative stance. In other words, institutionalized identity may not merely moderate the magnitude of the relationship between self-control and AI attitudes, but may—through reshaping the core psychological task individuals face when confronting AI—alter the functional direction in which self-control is mobilized. This does not mean that the emotional buffering function disappears in high-responsibility occupations; rather, risk vigilance gains higher functional priority in specific contexts. This functional shift does not imply that self-control loses adaptive value in high-responsibility settings. On the contrary, risk vigilance is an indispensable psychological foundation in high-reliability systems (Biedermann et al., 2024; Weick and Sutcliffe, 2015). The question is not whether self-control is “beneficial,” but that the goal it serves has changed—from facilitating individuals' emotional adaptation to new technology, to maintaining responsibility sensitivity toward system safety (Gao et al., 2021).

Social role theory provides support for this interpretation. Biddle (1986) noted that when individuals take on specific social roles, their cognition and judgment are profoundly shaped by role expectations and responsibility boundaries. For flight instructors, the role involves not only personal task execution but also responsibility for developing trainee capabilities and maintaining system safety (Fanjoy and Gao, 2011). These multiple responsibilities may amplify sensitivity to AI's potential risks, and this reasoning is descriptively consistent with the direction of the slope observed in the instructor group. We emphasize, however, that this positive slope is statistically non-significant and is based on a small subgroup (*n* = 34); accordingly, it should be treated as a preliminary, exploratory pattern that is at most suggestive of the proposed functional-shift account. Any confirmatory inference about a directional reversal in high-responsibility groups must await larger samples and ideally measures that directly tap the proposed psychological processes.

The supplementary analyses offer further interpretive evidence. All five self-control subscales significantly predicted negative AI attitudes in the student group, but none did so in the pilot or instructor groups. Moreover, three subscales related to immediate task execution—impulse control, focused work, and recreational moderation—showed significant group differences, whereas two subscales related to long-term lifestyle—healthy habits and resisting temptation—did not. These observations lead us to tentatively propose a functional differentiation framework for self-control: one cluster comprises “lifestyle-oriented” functions (e.g., healthy habits, resisting temptation), which are relatively stable and less susceptible to change with occupational role; the other comprises “task-execution-oriented” functions (e.g., impulse control, focused work, recreational moderation), which are more likely to be reconfigured in highly institutionalized occupational environments. This framework resonates with Fleeson and Jayawickreme's (2015) observations on the situational contextualization of trait functioning, though further research is needed to evaluate it.

Age is also worth noting. The three groups in this study differed substantially in age. Although the interaction effect remained significant after controlling for age, age may contribute to attitude formation through an additional pathway: older practitioners may be more concerned about “being left behind by technology,” and this concern—combined with heavier professional responsibilities—may amplify cautious attitudes. This speculation warrants further examination through more refined research designs in future work.

### 4.2 A proposed “Value–Dynamics” dual-dimensional framework: a conceptual extension for future research

Building on the empirical findings above, we now sketch a conceptual extension that may help future research dissect the heterogeneous psychological states that current single-dimensional measurement of AI attitudes tends to collapse together. We emphasize at the outset that the framework introduced in this section is not an empirical result of the present study: only the negative-evaluation dimension of AI attitudes was assessed (with the NARS), and the dynamics-orientation dimension—approach/use vs. avoidance/caution—was not directly measured. The framework should therefore be read as a theoretical proposal and a target for future empirical work that simultaneously assesses both dimensions, rather than as a structure derived from the present data. With this scope clearly delimited, we outline the framework below and use it to organize, but not to confirm, an interpretation of the group differences observed here.

Most AI attitude research operationalizes attitudes as a single continuum from positive to negative, implicitly assuming that “negative attitudes” primarily reflect tendencies to reject or avoid AI (Schepman and Rodway, 2020; Sindermann et al., 2021). In high-responsibility, high-risk occupational settings, however, this assumption may not hold. In domains such as aviation and automated systems, caution, reservations, and even concerns about AI do not necessarily reflect a rejection of the technology; they may instead stem from high sensitivity to safety, controllability, and the consequences of responsibility (Parasuraman and Riley, 1997; Weick and Sutcliffe, 2015). The conventional single-dimensional treatment of attitudes conceptually oversimplifies the complexity of the attitudinal structure.

Classical attitude theory has long recognized that attitudes are not unitary mental representations, but are composed of relatively independent components—cognitive appraisal, affective response, and behavioral intention—which may dissociate in specific contexts (Biddle, 1986; Fleeson and Jayawickreme, 2015). In AI attitude research, empirical findings have already shown that individuals can exhibit higher usage intentions or reliance tendencies at the functional level even while holding relatively negative affective or risk evaluations of AI (Gillath et al., 2021; Schaefer et al., 2016). Furthermore, the approach–avoidance framework in motivational psychology suggests that individuals can simultaneously experience both approach and avoidance motivational tensions toward the same target; these are not simple opposites, but can coexist and mutually constrain each other (Uziel and Baumeister, 2017). In high-risk decision-making or high-responsibility contexts, this coexistence of approach and avoidance is particularly pronounced, manifesting as simultaneously valuing the target while maintaining heightened vigilance toward its potential consequences (Biedermann et al., 2024; Warm et al., 2008).

**Figure 3:**
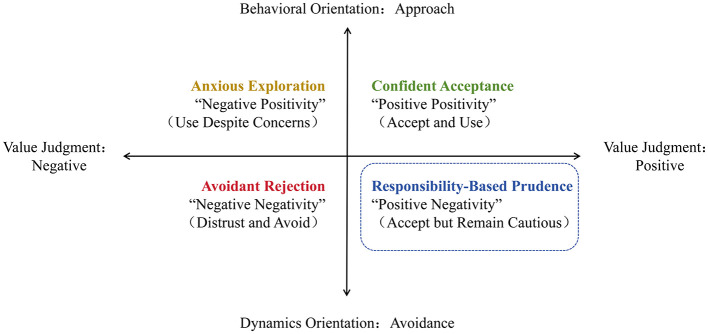
Dual-dimensional structural framework of AI attitudes.

Drawing on this theoretical integration, we propose—as a conceptual extension awaiting empirical validation—a “value–dynamics” dual-dimensional analytical framework for AI attitudes. The value-judgment dimension reflects individuals' overall cognitive appraisal of AI (positive vs. negative), primarily concerning perceptions of its utility, reliability, and risk. The dynamics-orientation dimension reflects behavioral tendencies in real contexts—approach/use or avoidance/caution. The two dimensions are relatively independent; their combination yields four meaningfully distinct AI attitude types (see Figure 3), which help to reveal psychological differences that a single-dimensional measurement approach would obscure, particularly in high-responsibility occupational settings.

**Confident acceptance** (positive valuation + approach orientation) refers to individuals who hold positive cognitive evaluations of AI and show stable, proactive usage intentions. This state corresponds to the ideal adoption pattern in TAM, typically accompanied by high technology self-efficacy and low uncertainty perception (Venkatesh et al., 2003). In this type, value judgment and behavioral orientation are highly consistent; individuals both recognize AI's functional value and possess the psychological readiness to incorporate it into practice.

**Anxious exploration** (negative or ambivalent valuation + approach orientation) refers to individuals who, despite harboring concerns or ambivalent evaluations of AI, still exhibit tendencies toward engagement and use. This dissociation between evaluation and motivation has been documented in technology anxiety research: individuals may recognize the necessity and inescapability of a technology and thus choose to approach and try it, yet experience significant anxiety due to uncertainty about their own capabilities or technology consequences (Meuter et al., 2003). The present study found that among university students, self-control was significantly negatively correlated with negative AI attitudes—the higher the self-control, the lower the reported negative attitudes. Within this framework, this pattern would be consistent with the possibility that “negative attitudes” in the student sample may to a large extent reflect anxiety responses to novel intelligent technology rather than stable value rejection. On this interpretation, self-control would operate primarily as a regulator of anxiety, thereby reducing the defensive evaluations induced by capability uncertainty. In other words, such negative attitudes more closely approximate a temporarily protective psychological response than a genuine rejection of AI.

**Responsibility-based prudence** (positive or neutral valuation + cautious orientation) refers to individuals who cognitively recognize AI's functional value but maintain cautious, restrained, and continuously monitoring behavioral orientations in its application. This attitude type is regarded as an adaptive psychological response in high-reliability organization research: in safety-critical systems, maintaining vigilance and limited trust toward automation is an important prerequisite for stable system functioning (Biedermann et al., 2024; Weick and Sutcliffe, 2015). The present finding that the relationship between self-control and negative AI attitudes is no longer significant among pilots, and even trends positive among flight instructors, forms a sharp contrast with anxious exploration. Within the proposed framework, this pattern would be consistent with the interpretation that higher “negative attitudes” in high-responsibility professional groups may not primarily stem from anxiety or capability uncertainty, but may instead reflect rational prudence grounded in responsibility consciousness and risk evaluation. Under this interpretation, self-control's role would not be to buffer negative emotions but to help individuals maintain sensitivity to potential risks, thereby avoiding over-reliance on automated systems (Parasuraman and Riley, 1997).

**Avoidant rejection** (negative valuation + avoidance orientation) refers to individuals who hold stable negative cognitive evaluations of AI and exhibit clear avoidance or rejection tendencies in behavior. This type closely parallels patterns described in algorithm aversion research, whereby individuals form persistent distrust of AI after perceiving algorithmic errors, opacity, or uncontrollable risk, and actively avoid its use (Dietvorst et al., 2015). Compared with the previous types, the value judgment and behavioral orientation here point in the same direction; the negative attitudes in this type are more likely to reflect stable belief structures rather than situational anxiety or responsibility-driven prudence.

The core insight of this framework is that seemingly similar “negative attitudes” may correspond to fundamentally different psychological states. Anxious exploration and responsibility-based prudence may yield similar scores on a negative attitudes scale, yet the negativity of the former stems from capability anxiety while that of the latter stems from responsibility vigilance—and their underlying psychological mechanisms and intervention needs are entirely different. For the anxiously exploring group, intervention should focus on enhancing technology self-efficacy and reducing capability anxiety. For the responsibility-based prudence group, simply “eliminating resistance” is not the appropriate direction; what matters more is clarifying human–machine division of labor and responsibility boundaries through institutional design, so that cautious attitudes become a guarantee of system safety rather than an obstacle.

It should be noted that the present study measured only the negative dimension of attitudes and did not directly measure dynamics orientation. The foregoing framework is a theoretical extrapolation based on the research findings; its validity awaits future research that simultaneously assesses both dimensions.

### 4.3 Contributions, limitations, and future directions

The core contribution of this study is providing empirical evidence on the situational boundary of psychological trait effects. For a long time, trait research has implicitly assumed cross-situational consistency; the present finding—that the relationship between self-control and AI attitudes manifests in markedly different patterns across institutionalized identities—directly challenges that assumption. Building on this, the study proposes two explanatory frameworks: a situational-shift framework for self-control's functional priority, distinguishing “emotional buffering” from “risk vigilance”; and a “value–dynamics” dual-dimensional framework for AI attitudes, offering conceptual tools for understanding the heterogeneous psychological states underlying “negative attitudes” in different groups.

The conclusions of this study need to be understood within certain boundary conditions. In terms of sampling, the three groups were unequal in size; the instructor group comprised only 34 participants, and although the positive trend observed in that group is consistent with theoretical expectations, statistical power is limited and further verification with larger samples is needed. Moreover, age and institutionalized identity co-varied in this study; although the interaction effect remained significant after controlling for age, their independent contributions await further disentanglement through age-matched or longitudinal designs. Additionally, gender distribution differed substantially across groups—female participants constituted 65.4% of the student sample, whereas pilots and flight instructors were predominantly male, reflecting the actual demographic composition of the aviation industry. Although gender was statistically controlled in the robustness analysis alongside age, and the core interaction effect remained significant, the pronounced gender imbalance across groups represents a potential confound that future studies should address through more balanced sampling designs or targeted statistical controls. In terms of measurement, this study focused on the negative dimension of attitudes, and the dynamics-orientation dimension of the “value–dynamics” framework has not been directly measured; the framework's validity awaits future empirical testing. Additionally, the proposed psychological processes underlying the observed group differences—capability anxiety in the student group and responsibility-based vigilance in the high-responsibility groups—were not directly measured in this study. The “functional-shift” account should therefore be read as a plausible interpretation of the present pattern of results, awaiting direct empirical testing through measures that tap state anxiety, perceived professional responsibility, and vigilance demands. A further important limitation concerns the measurement of AI experience. We assessed only organizational AI exposure (whether the respondent's institution had adopted AI-related tools) and did not measure individual frequency or intensity of AI use. Because AI usage frequency may itself shape AI attitudes—and may differ systematically within groups (e.g., between undergraduate and postgraduate students)—its omission is a genuine limitation of the present design rather than merely a direction for future elaboration. Organizational exposure was, moreover, strongly confounded with occupational group (*r* = −0.56); although the interaction remained significant when it was included as a covariate, future studies should incorporate validated, graded measures of individual AI usage frequency and type. In terms of generalizability, the sample was drawn from the aviation sector; whether the conclusions extend to other high-responsibility domains such as healthcare or nuclear energy requires cross-industry comparative research.

These limitations also point to several directions worthy of deeper investigation: Do the subdimensions of self-control exhibit functional differentiation across different occupational contexts? Do similar psychological mechanisms exist in other high-reliability organizations beyond aviation? How can measurement instruments be designed to simultaneously capture both value judgment and dynamics orientation? Addressing these questions will help further delineate the situational boundaries of the relationship between psychological traits and technology attitudes.

## 5 Conclusion

The present study found that the relationship between self-control and negative AI attitudes appears to vary by institutionalized identity. Among university students, self-control was negatively associated with negative AI attitudes; among pilots, this association was no longer statistically significant; and among flight instructors, a non-significant positive trend emerged that—given the small subgroup size (*n* = 34)—is offered as a preliminary, exploratory pattern rather than a confirmed effect. These findings are consistent with the broader interpretation that the attitudinal effects of psychological traits may have situational boundaries, and that their functional meaning may depend on the responsibility structure and occupational role in which the individual is embedded.

Building on this empirical pattern, the study offers two conceptual contributions intended to guide future research rather than to summarize directly tested mechanisms: a situated functional-shift account, in which self-control may transition from an “emotional buffering” function to a “risk vigilance” function as institutionalized identity deepens; and a “value–dynamics” dual-dimensional analytical framework for AI attitudes, presented as a theoretical extension awaiting empirical validation that simultaneously assesses both dimensions. Taken together, the empirical results and these proposals suggest that psychological trait effects on technology attitudes may be bounded by occupational responsibility structures, and that effective AI governance and professional training in high-responsibility industries would benefit from being designed with sensitivity to the distinct psychological orientations of different occupational groups rather than applying universal assumptions derived from general-population research.

## Data Availability

The datasets presented in this article are not readily available because: the dataset used in this study contains confidential information from professional aviation practitioners (pilots and flight instructors) and is protected by privacy and ethical agreements. Due to privacy and confidentiality agreements, professional confidentiality requirements, and participant privacy protection protocols, the raw data cannot be made publicly available. Access to the dataset may be considered only for legitimate research purposes upon reasonable request to the corresponding author, with approval from the institutional ethics committee of Shaanxi Normal University. Requests to access the datasets should be directed to Ying Long, longying@snnu.edu.cn.
